# Clinical-oriented tacrolimus dosing algorithms in kidney transplant based on genetic algorithm and deep forest

**DOI:** 10.3389/fphar.2025.1656197

**Published:** 2025-08-29

**Authors:** Jianliang Min, Qihao Li, Weijie Lai, Yingqi Lu, Xintong Wang, Guodong Chen

**Affiliations:** ^1^ Organ Transplantation Center, The First Affiliated Hospital, Sun Yat-sen University, Guangzhou, China; ^2^ School of Medicine, Jiaying University, Meizhou, China; ^3^ Guangdong Provincial Key Laboratory of Organ Medicine, The First Affiliated Hospital, Sun Yat-sen University, Guangzhou, China; ^4^ Guangdong Provincial International Cooperation Base of Science and Technology (Organ Transplantation), The First Affiliated Hospital, Sun Yat-sen University, Guangzhou, China; ^5^ Guangdong Provincial Clinical Research Center for Cancer, Sun Yat-sen University Cancer Center, Guangzhou, China; ^6^ State Key Laboratory of Oncology in South China, Sun Yat-sen University Cancer Center, Guangzhou, China

**Keywords:** kidney transplant, tacrolimus, genetic algorithm, deep forest, personalized dosing, machine learning

## Abstract

**Background:**

The immunosuppressant tacrolimus (TAC) plays a crucial role in preventing rejection reactions after organ transplant. Due to a narrow therapeutic window, it is one of the long-term challenges in postoperative care, increasingly requiring a precise management due to individual variability. To alleviate the burden on clinicians and achieve an automatic and precise drug dosing, the AI-assisted personalized dosing of TAC is a promising predictive method.

**Methods:**

This study presents a clinical-oriented TAC dosing algorithm that integrates genetic algorithm (GA) with deep forest (DF) to predict both initial and follow-up doses for kidney transplant recipients. The optimized candidate variables were first conducted from numerous clinical factors by GA using support vector regression based on radial basis function. Then a smaller number of key clinical variables were confirmed for clinical relevance and ease of use by an exhaustive feature selection method.

**Results:**

Validated in a cohort of 288 recipients, the DF model combined with a few clinical variables ultimately achieved an average accuracy of 84.5% and 91.7% in the initial and follow-up dosage prediction.

**Conclusion:**

The proposed approach can provide a potential reference to algorithm-based automatic pipeline methods for drug dosing prediction and analysis in clinical practice.

## 1 Introduction

Chronic kidney disease is a common progressive disease which can lead to the gradual irreversible decline or even loss of kidney function (Kovesdy, 2022). Global Burden of Disease (GBD) research indicates that it has become a leading cause of death worldwide (Abdalla et al., 2015). The end stage of chronic renal dysfunction is uremia. Kidney transplant is now recognized as the best treatment for uremia (Axelrod et al., 2018). However, immunosuppressive therapy after operation is key to maintaining renal function and the long-term effect of renal transplant. As a first-line immunosuppressant, tacrolimus (TAC) not only demonstrates a lower incidence of acute rejection but also a reduced risk of graft failure than cyclosporine (CsA) (Vincenti et al., 2002). The combination of TAC with mycophenolate mofetil (MMF) and glucocorticoid has become a classic anti-rejection therapy in organ transplant.

The main challenge in the rational use of TAC in clinical settings concerns its narrow therapeutic window and large individual variability in pharmacokinetics (Schagen et al., 2023). For recipients, the low blood trough concentration (C_0_) of TAC may cause graft rejection, while the high C_0_ may cause an adverse drug reaction. For careful dosing, therapeutic drug monitoring (TDM) is implemented in the recommended clinical guidelines (Brunet et al., 2019). Nonetheless, many transplant recipients still experience suboptimal TAC exposure (under-/overexposure), resulting in the decline of graft survival. Amid growing demands for personalized drug delivery and precision medicine, traditional TAC dosing regimens based on limited clinical parameters such as body weight and prior C_0_ face an increasing challenge (Radhakrishnan et al., 2021).

A current remarkable technique is based on artificial intelligence algorithms (Hamet and Tremblay, 2017). The increasing abundance of clinical, genetic, radiological and metabolic data in transplantation has led to a growing interest in applying machine learning algorithms. On this basis, dosing-related algorithms have been developed to make individualized dosing more efficacious and safe while reducing toxicity and adverse events (Diez-Sanmartin et al., 2021). Initial and follow-up dose prediction in early studies rely on pharmacokinetic modeling, with population pharmacokinetic (popPK) being the principal method (Schagen et al., 2023; Andrews et al., 2018). For example, Zhu et al. (2018) developed a popPK model by using different genotypes, such as CYP3A5/3A4/POR, to predict the dose of TAC. These models required specialized pharmacokinetic knowledge, rendering them relatively complex for clinical usage (Staatz et al., 2002). In addition, traditional models such as linear regression (LR), random forest (RF), support vector regression (SVR), and multi-model comparison have been often used in forecasting TAC doses (Stifft et al., 2020; Van Looy et al., 2007; Cai et al., 2020; Tang et al., 2017; Zhang et al., 2022). However, these studies exhibited wide variation in their findings, and the interpretability of models based on available clinical variables has remained limited. Furthermore, the pharmacokinetics of TAC are influenced by factors including patient demographics, genetic polymorphisms, postoperative time, drug combination, hemoglobin (HB), and alanine aminotransferase (ALT) (Kirubakaran et al., 2020; Stratta et al., 2012). Therefore, strategies to confirm and synthesize these factors require further investigation to avoid model designs burdened by excessive variables or poor clinical applicability.

**FIGURE 1:**
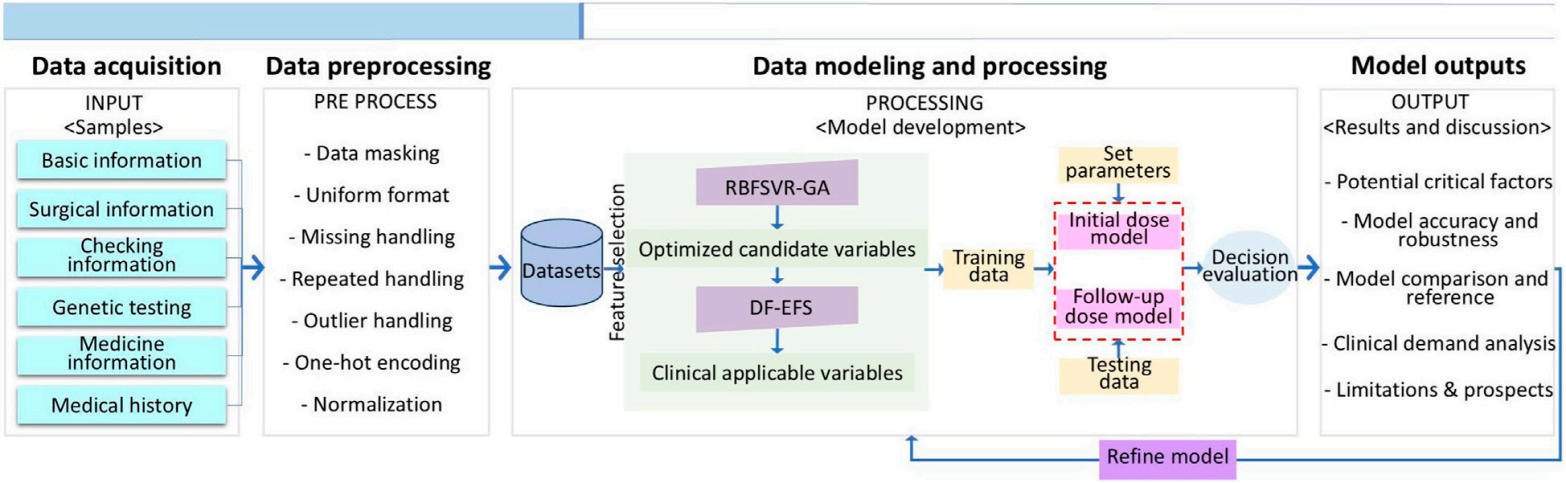
Flowchart of the GA-DF based TAC dose prediction process.

This study aims to develop a clinically oriented prediction pipeline for accurately determining initial and follow-up doses of TAC in kidney transplant recipients. We investigated whether key factors derived from extensive demographic, clinical, genetic, and metabolic data could effectively predict dosing requirements. The key contribution is a clinically applicable pipeline that achieves significant predictive accuracy with minimal variables. A feature optimization by GA was first applied to identify candidate variables—a commonly robust and powerful heuristic search algorithm (Leardi et al., 1992). Then a smaller range of variables was surveyed by a histogram statistic of exhaustive feature selection (EFS) combined with expert opinions. Dose prediction using DF was carried out—an ensemble learning method selected for its efficacy and simplicity. Results demonstrated that the GA-DF algorithm provides potential benefit for drug-dosage decisions in relevant areas and may have a complementary role in existing methods. Figure 1 illustrates the workflow encompassing data acquisition, preprocessing, feature selection, model optimization, and outcome analysis.

## 2 Materials and methods

### 2.1 Study patient

**FIGURE 2:**
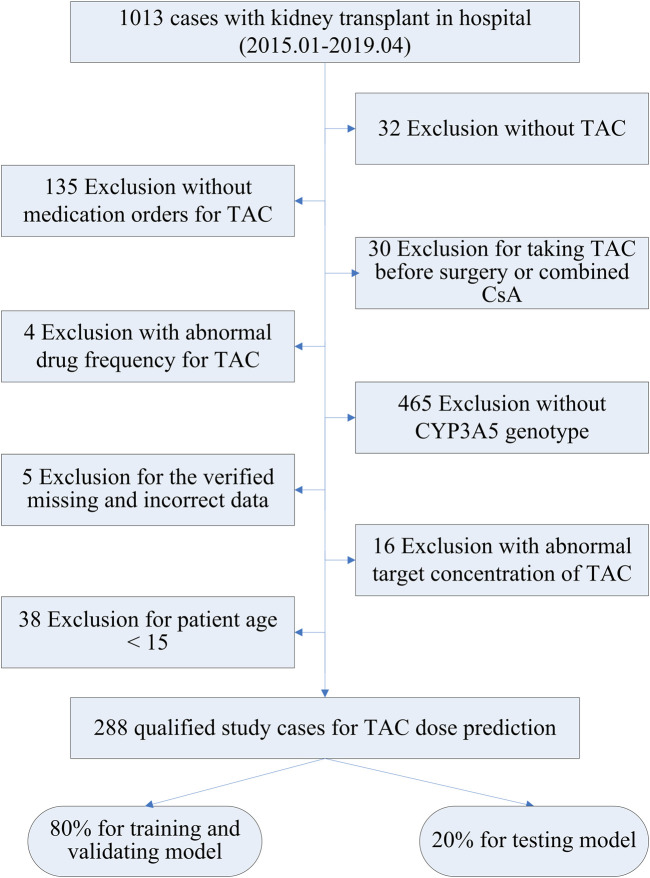
Snapshot of data collection for kidney transplant.

The records of 1013 Chinese kidney transplant patients at the First Affiliated Hospital of Sun Yat-sen University between January 2015 and April 2019 were retrospectively collected. Eligible patients received TAC as part of an immunosuppressive regimen after transplant, and took the TAC drug twice daily, every 12 h. The detailed enrollment of patients is displayed in Figure 2. Exclusions included 465 patients lacking CYP3A5 genotype data and 135 failing to adhere to TAC medication instructions (dosage, frequency, record), accounting for 82.8% of all exclusions. A further 62 patients were excluded for not taking TAC, preoperative use, or concomitant CsA. A small number of samples with abnormal records or missing data were not considered. The current analysis also excluded TAC use in pediatric patients, given the limited sample size and their significant differences from adults. Ultimately, 288 qualified study patients with TAC daily doses were enrolled, 80% of whom randomly served as the training cohort and 20% as the testing cohort. All studies were conducted in accordance with the Declaration of Helsinki and Good Clinical Practice, using exclusively authorized clinical datasets proprietary to the First Affiliated Hospital of Sun Yat-sen University. The study protocol was approved by the hospital’s ethics committee (No. 2022–166).

### 2.2 Data acquisition and processing

Collected variables included demographics (age, sex, and weight), surgical details (surgery type/time, induction therapy), biochemical data, metabolic genotypes, medication records, and medical history such as diabetes. Specifically, the genotypes refer to three types: CYP3A5*1/*1, CYP3A5*1/*3, and CYP3A5*3/*3. Biochemical data involved common indicators such as HB, ALT, hematocrit (HCT), creatinine (CR), total bilirubin (TB), red blood cells (RBC), albumin (ALB), and aspartate aminotransferase (AST). These indicators can reflect common postoperative complications (e.g., hepatotoxicity, nephrotoxicity, and anemia), helping differentiate whether abnormal TAC concentrations result from metabolic alteration or organ dysfunction and thereby guiding personalized dose prediction (Staatz and Tett, 2004). For the last preoperative parameter, we added the prefix “pre”; for the first postoperative measure, we added the prefix “pos”. For example, preHCT/posHCT respectively express the last preoperative/first postoperative value of HCT. For laboratory values measured at the time point closest to TAC dosing, the suffix “_NN” was added. Table 1 presents all original variables included in the initial and follow-up dose prediction (M1 and M2). In addition, the TAC-C_0_ target used after transplantation was set at 5–10 ng/mL in our department (Otabil, 2019). All recorded data had a unified format by removing spaces, abnormal characters, and repeated/outlier values. Nearest neighbor interpolation for missing data and one-hot encoding for categorical variables were used. Finally, the features were normalized by adopting min–max normalization for convergence and efficiency (Patro and Sahu, 2015).

**TABLE 1:** Original variables for initial/follow-up dose prediction.

Models	Original variables
Initial dose prediction	Age, sex, weight, surgType, CYP3A5, diabetes, GCs, PPI, CCB, MMF, preHB, preHCT, preCR, preTB, preALB, preALT, preAST, posHB, posHCT, posCR, posTB, posRBC, posALB, posALT, posAST
Follow-up dose prediction	Age, sex, weight, surgType, CYP3A5, diabetes, MMF, priDose, priDose_surgTimeD, priC_0_, priC_0__surgTimeD, HB_NN, HCT_NN, CR_NN, TB_NN, RBC_NN, ALB_NN, ALT_NN, AST_NN

surgType, surgery type; GCs, glucocorticoids; PPI, proton pump inhibitor; CCB, calcium channel blocker; MMF, mycophenolate mofetil; RBC, red blood cells; ALB, albumin; HB, hemoglobin; HCT, hematocrit; CR, creatinine; TB, total bilirubin; ALT, alanine aminotransferase; AST, aspartate aminotransferase; priDose, prior TAC dose; priDose_surgTimeD, time interval of prior dose after surgery; priC_0_, prior trough concentration; priC_0__surgTimeD, time interval of prior C_0_ after surgery; CYP3A5, genotype; pre, last preoperative; pos, first postoperative; _NN, nearest neighbor.

### 2.3 Variable selection

The primary purpose of feature selection is to identify a more compact set of clinically applicable variables while minimizing model performance degradation. The application of GA is a potentially robust and effective optimization method (Leardi et al., 1992). It is a heuristic computing model that simulates the natural evolution process to reach a global optimal solution. GA can effectively identify the most distinguishable features, which is very useful for the preliminary selection of potential candidate variables. It follows the following steps: (i) initializing population, randomly generating *pop_size* from the feature space, and representing each individual as a binary encoding; (ii) assessing the fitness of each individual in the population by a fitness function; (iii) selecting parental individuals according to fitness by setting the individual number *tournsize*; (iv) performing crossover on the selected parent to generate new individuals with the *cxpb* parameter and replace the original parents; (v) mutating certain individuals in the new population with the *mutpb* parameter; (vi) evaluating the fitness of newly generated individuals to replace the low fitness of individuals in the original population; (vii) repeating steps iii ∼ vi and gradually evolving the population until there is a satisfactory value or the maximal number of iterations is reached by parameter *ngen*.

For strong stability, SVR with the radial basis function (RBF) was used as the fitness function. The RBF kernel can inherently mitigate feature collinearity effects. Furthermore, the fitness function incorporates a collinearity penalty term, formulated as a weighted sum of prediction error and collinearity index to explicitly address multicollinearity. The GA toolbox was achieved by using the public DEAP library of Python (deap 1.4.1 available at https://pypi.org/project/deap/). Its core parameters are listed in Table 2 by a grid search (Liashchynskyi and Liashchynskyi, 2019), while other parameters were default. In addition, EFS is a simple greedy algorithm which can provide an optimal feature combination. This approach enables the extraction of fewer key clinical variables from optimized candidate pools. The results of all combinations were calculated ten times to smooth the randomness of tree-based models. Then a histogram statistic was used to identify the final key clinical factors.

**TABLE 2:** Parameters of the algorithms used.

Algorithms	Core parameters
GA	SVR: kernel = 'rbf’; cv = 10; param_grid # ‘C': [0.001, 0.01, 0.1, 1, 10, 100], ‘gamma’: ['scale']; tournsize = 3 pop_size: 100 cxpb: 0.70 mutpb: 0.05 ngen: 200 criterion: -mse
DF	n_estimators: 8 max_layers: 20 n_trees: 100 criterion: mse

### 2.4 Algorithm configuration

The DF model has unique structure and characteristics (Zhou and Feng, 2019). Unlike traditional deep learning methods, it does not require a large number of labeled data and features due to its nature as a powerful tree-based ensemble approach. It is particularly suitable for a small amount of clinical data in structured medical data. Moreover, it performs better than existing tree-based ensemble methods such as AdaBoost (Freund and Schapire, 1996) and LightGBM (Ke, 2017), with faster-training speed and a higher efficiency. Therefore, the DF algorithm was adopted to predict TAC dosages, achieving excellent performance with a manageable cost for both variable selection and regression analysis. To our knowledge, the application of cascaded deep forest with ensemble learning for TAC dosage prediction has not been previously reported in the literature.

**FIGURE 3:**
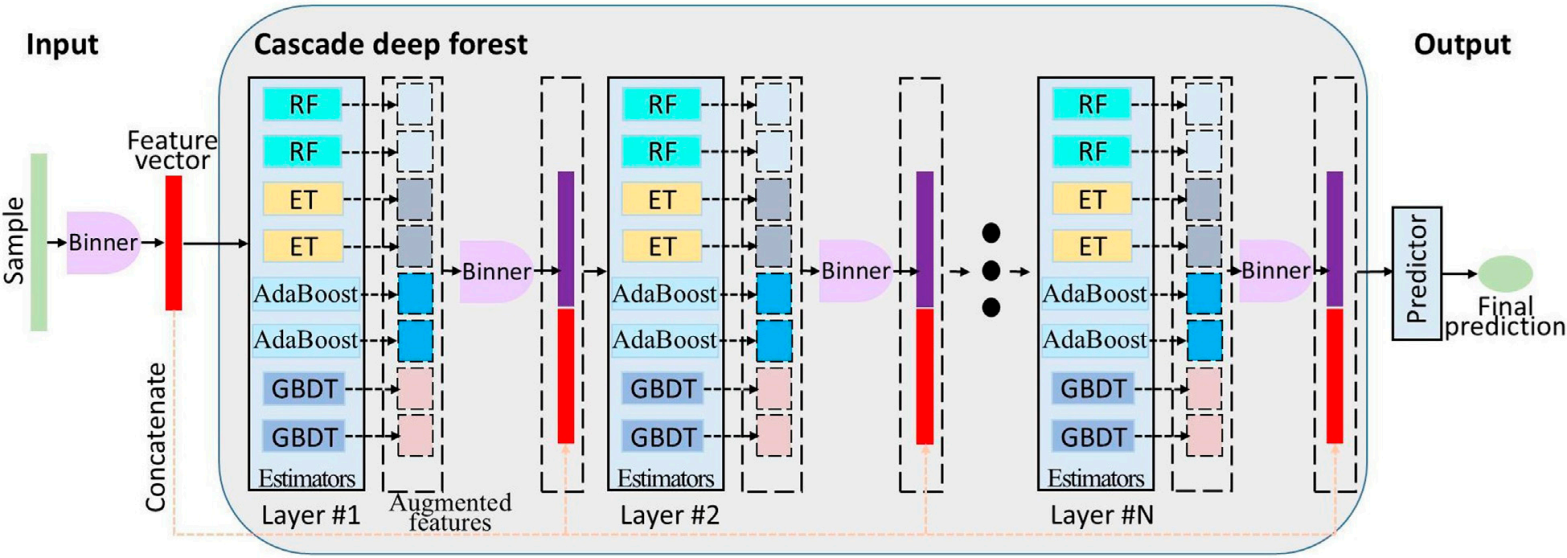
Schematic of the deep forest framework. At the core of this model are eight heterogeneous estimators: two RF, two ET, two AdaBoost, and two GBDT base learners. Different layers have the same structure.

Figure 3 depicts the multilayer architecture of DF, where each layer consists of multiple estimators. These estimators learn feature information from the input feature vector and pass the processed information to the next layer. Each layer owns various types of estimators to enhance the robustness of model. Binner was first used to reduce the number of splitting candidates for building decision trees. Subsequently, the initial cascade layer of DF was constructed using binned data. This layer integrated eight estimators: two RF, two ET (Geurts et al., 2006), two AdaBoost, and two GBDT (Friedman, 2001). Furthermore, it concatenates the augmented features to the binned training samples, serving as the new training data for next layer. Until the last cascade layer, predictions from constituent learners are aggregated to generate the final output. In addition, the number of cascade layers can be adaptively determined by data complexity and sample size. The DF package (version 0.1.7) is publicly available at https://pypi.org/project/deep-forest/. Core parameters are also documented in Table 2.

### 2.5 Performance metrics

To evaluate and compare the model’s performance, accuracy (Acc), mean square error (MSE), mean absolute error (MAE), median absolute error (MEAE), max error (ME), and fitting index of *R* ^2^ were utilized. Acc is defined as the proportion of predictions satisfying |ŷ - y| ≤ 1.2 mg, where ŷ denotes the predicted TAC dose and y represents the actual dose. This threshold was set based on the minimum TAC dosage unit of 0.5 mg within clinically acceptable limits in our hospital. MSE is the average of the sum of the square difference, and MAE is the average of the absolute difference both between ŷ and y. MEAE measures the median of the absolute difference, and ME describes the maximum absolute difference both between ŷ and y. *R* ^2^ is the squared correlation between ŷ and y where the closer it is to 1, the better the fitness.

For statistics, the significance of the adjacent Acc in the histogram was determined by Wilcoxon rank-sum test. The pre-analysis used *t* tests to compare the differences between the training and testing cohorts, including age, sex, weight, CYP3A5 genotype, and biochemical parameters. The median and interquartile range (IQR) was used to describe the distribution of continuous variables. To enhance the clinical interpretability of model, we employed Shapley additive explanations (SHAP) analysis (Choshi et al., 2025). This game theory-based method quantifies feature-specific contributions while maintaining consistency with model outputs. SHAP was selected for its ability to provide the global feature importance ranking and generate clinical insights into prediction explanations. It is a very popular method for interpreting the prediction of any machine learning algorithm (Lundberg and Lee, 2017) and is available at https://pypi.org/project/shap/ (shap 0.42.1).

### 2.6 Calculation tool

All analyses were conducted in Python 3.7.1 using the Spyder 3.3.2 scientific environment. The scikit-learn library implemented core machine learning workflows including data splitting, hyperparameter tuning via grid search, feature scaling, and model evaluation (Pedregosa et al., 2011). Additionally, we implemented five regression modules by scikit-learn: LR via LinearRegression; RF via RandomForestRegressor; ET via ExtraTreesRegressor; AdaBoost via AdaBoostRegressor; GBDT via GradientBoostingRegressor. SVR was used via sklearn. The svm.SVR module with the parameter *gamma = ‘scale’* and *C* was confirmed via grid search over [0.01, 0.1, 1, 10, 100, 1000]. LightGBM was executed through lightgbm. LGBMRegressor (v4.3.0 available at https://pypi.org/project/lightgbm/). All experiments were conducted by a computer with a 12th Gen Intel(R) Core (TM) i7-12700F@2.10 GHz CPU and 16 GB RAM.

## 3 Results

### 3.1 Basic characteristics and pre-analysis

The cohort included 288 patient records. The basic characteristics of patients are presented in Table 3. Continuous variables are expressed as median (IQR) and categorical variables as number (%). The distribution of age and weight was 37.0 (30.0–50.0) and 56.0 (49.5–63.5), respectively for a thin, middle-aged transplant recipient population. Most were men with 206 cases (71.5%). In these patients, the daily dose of TAC was 6 (5–7) mg while the related C_0_ was 9.8 (4.9–13.6) ng/mL. Most patients (216, 75.0%) underwent deceased donor kidney transplantation. Additionally, the distribution of CYP3A5 genotypes was as follows: *1/*1 accounted for 10.8%, *1/*3 for 42.3%, and *3/*3 for 46.9%. The *1/*3 and *3/*3 genotypes were predominant in the Chinese population. Other variables in the table involved common biochemical data such as HCT, CR, ALT, and AST. The remaining variable types are Boolean values such as diabetes.

**TABLE 3:** Basic characteristics of patients

Variables	Whole cohorts (n = 288)
Continuous variable, median (IQR)	
Age (year)	37.0 (30.0–50.0)
Weight (kg)	56.0 (49.5–63.5)
GCs (mg)	27.9 (27.1–28.8)
MMF (g)	3.0 (3.0–4.0)
HB (g/L)	97.8 (83.0–114.0)
HCT (L/L)	0.31 (0.25–0.38)
CR (umol/L)	431.0 (180.8–883.3)
TB (umol/L)	11.5 (8.3–16.1)
RBC (*10^12^/L)	3.3 (2.8–3.8)
ALB (g/L)	38.5 (34.4–42.9)
ALT (U/L)	24.3 (11.4–47.5)
AST (U/L)	21.5 (13.6–38.6)
Dose (mg)	6 (5–7)
C_0_ (ng/mL)	9.8 (4.9–13.6)
Categorical variable, n (%)	
Sex	
Male	206 (71.5%)
Female	82 (28.5%)
Surgery type	
Living	72 (25.0%)
Allograft	216 (75.0%)
CYP3A5	
*1/*1	31 (10.8%)
*1/*3	122 (42.3%)
*3/*3	135 (46.9%)
Diabetes	
Yes	31 (10.8%)
No	257 (89.2%)
Hypertension	
Yes	158 (54.9%)
No	130 (45.1%)

GCs, glucocorticoids; MMF, mycophenolate mofetil; HB, hemoglobin; HCT, hematocrit; CR, creatinine; TB, total bilirubin; RBC, red blood cells; ALB, albumin; ALT, alanine aminotransferase; AST, aspartate aminotransferase; C_0_, blood trough concentration; CYP3A5, genotype.

The two sub-cohorts exhibited similar demographic profiles. In the training cohort, the mean patient age was 40.3 years, with an average weight of 57.8 kg; 26.1% of recipients were women. Similarly, the testing cohort comprised patients (mean age: 38.2 years) with a lower average weight (54.2 kg) and a higher proportion of women (37.9%). There were no significant differences between two groups allocated to different datasets in terms of age (*t* = 1.14, *p* = 0.26), sex (*t* = 1.68, *p* = 0.10), or body weight (*t* = 1.84, *p* = 0.07). For genotype, CYP3A5, *1/*1 accounted for 9.1%, *1/*3 for 42.2%, and *3/*3 for 48.7% in the training cohort, while the testing cohort contained 17.2%, 43.1%, and 39.7%, respectively. Moreover, no statistically significant difference was observed in genotypes between the two groups (*t* = 1.64, *p* = 0.10). Likewise, the groups did not differ in their biochemical data by independent testing.

### 3.2 Number screening in candidate variables

**FIGURE 4:**
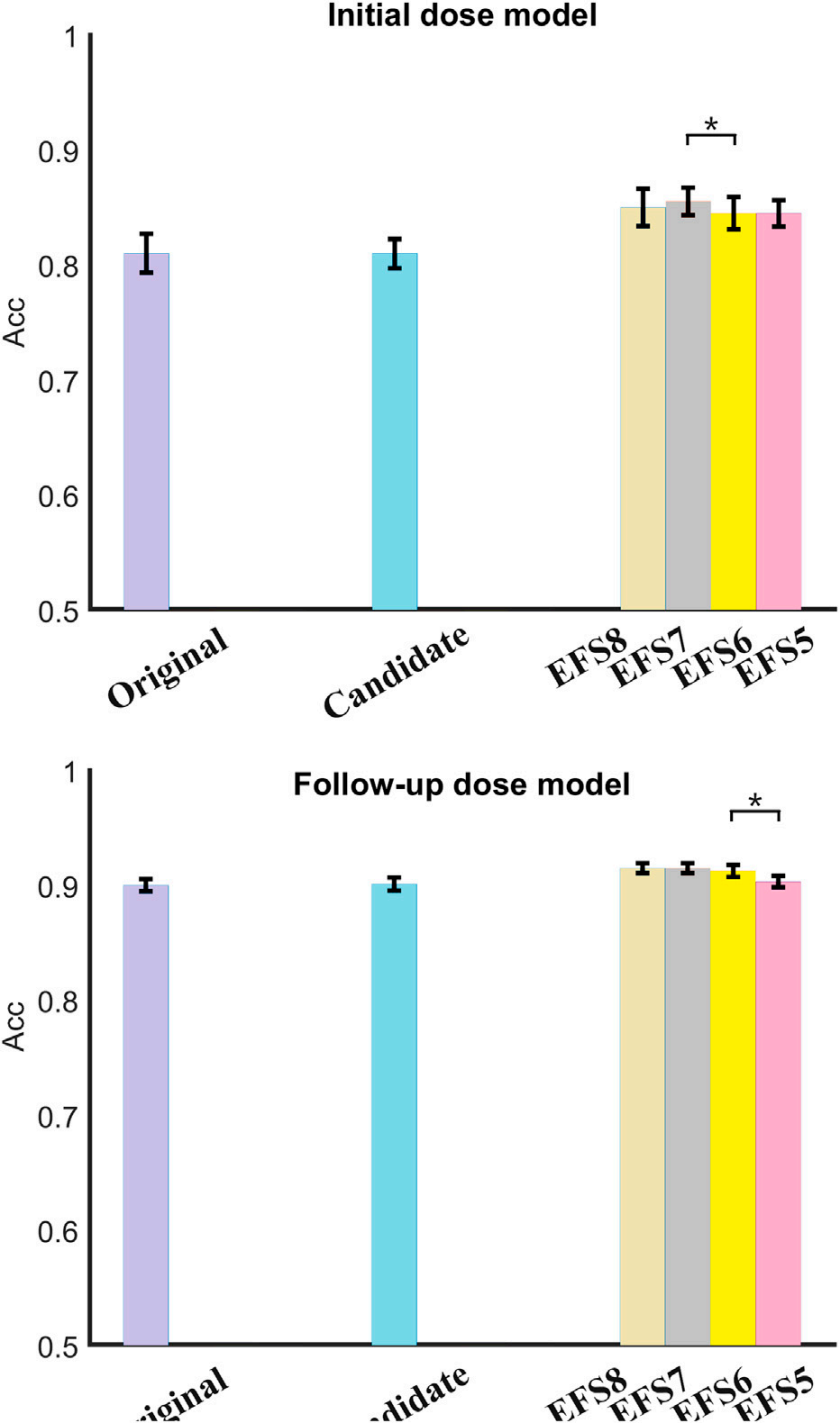
Comparison of variable sets in both models (*p < 0.05). Significant differences emerged during feature reduction: in initial dose prediction, between seven and six variables (p = 0.04), and in follow-up dose prediction between six and five variables (p = 0.03). Variable combination was obtained by EFS. For example, EFS8 denotes selection of the optimal eight factors from candidate variables using EFS. The others can be inferred by analogy.

Table 4 lists the results of optimized candidate variables for starting and follow-up dose prediction. The selected variables were consistent with clinical experience in TAC and were further analyzed in the discussion. Furthermore, the candidate variables were reused by EFS to confirm the optimal number of factors. Figure 4 compares variable sets of different sizes. The result represents the mean accuracy of predicted doses with the standard deviation. It is evident that there is no significant difference between the results of original and candidate variables, with the former achieving 81.0% (±0.017) and the latter 81.0% (±0.013) in M1, while the former are 90.0% (±0.005) and the latter 90.1% (±0.006) in M2. Both M1 and M2 showed improved performance with fewer variables, indicating redundant features in the original data. Specifically, no significant differences were observed when comparing eight versus seven variables in either M1 or M2. Similarly, reducing variables from six to five in M1 and from seven to six in M2 showed no significant performance changes. However, significant differences were observed when reducing features: in M1 between seven and six variables (*p* = 0.04 < 0.05), and in M2 between six and five variables (*p* = 0.03 < 0.05). The results of M1 using different variable numbers were 85.0% (±0.016), 85.7% (±0.012), 84.4% (±0.014), and 84.4% (±0.012) in turn, while those of M2 were 91.5% (±0.004), 91.5% (±0.004), 91.3% (±0.005), and 90.3% (±0.005), respectively. This suggests that the optimal feature number of M1 and M2 was seven and six in the final algorithm.

**TABLE 4:** RBFSVR-GA-optimized variables in both models.

Models	Candidate variables
Initial dose prediction	Age, sex, weight, preCR, preALT, preAST, posRBC, posHB, posTB, MMF, surgType, CYP3A5
Follow-up dose prediction	priDose, priC_0_, weight, diabetes, posALB, posTB, ALB_NN, HB_NN, CR_NN, TB_NN, CYP3A5

preCR, last preoperative creatinine; preALT, last preoperative alanine aminotransferase; preAST, last preoperative aspartate aminotransferase; posRBC, first postoperative red blood cells; posHB, first postoperative hemoglobin; posTB, first postoperative total bilirubin; MMF, mycophenolate mofetil; surgType, surgery type; priDose, prior TAC, dose; priC_0_, prior trough concentration; posALB, first postoperative albumin; ALB_NN, nearest neighbor albumin; HB_NN, nearest neighbor hemoglobin; CR_NN, nearest neighbor creatinine; TB_NN, nearest neighbor total bilirubin; CYP3A5, genotype.

### 3.3 Clinically applicable variables of models

**FIGURE 5:**
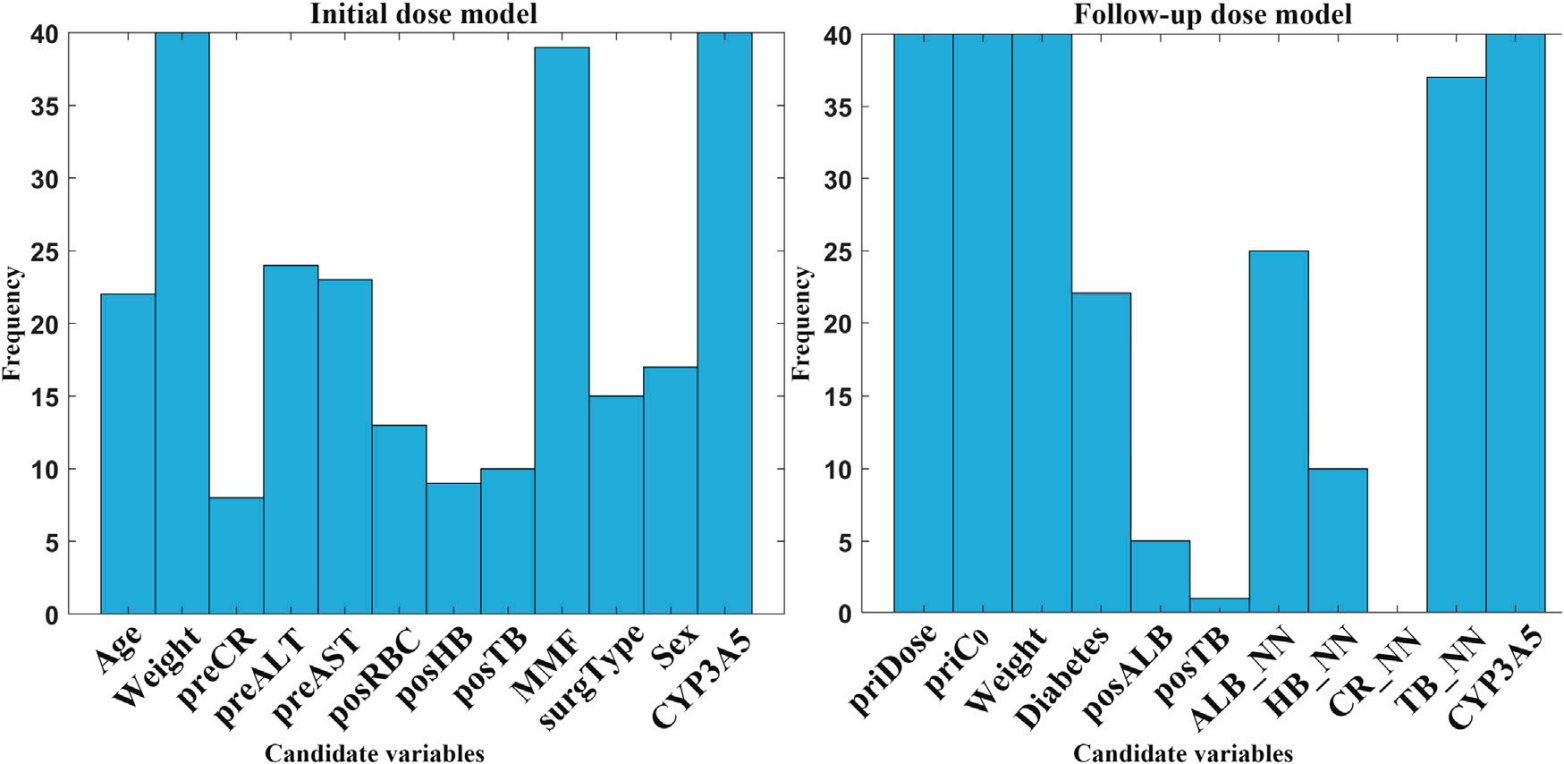
Frequency histogram of candidate variables selected by RBFSVR-GA across all combinations in both models. (preCR, last preoperative creatinine; preALT, last preoperative alanine aminotransferase; preAST, last preoperative aspartate aminotransferase; posRBC, first postoperative red blood cells; posHB, first postoperative hemoglobin; posTB, first postoperative total bilirubin; MMF, mycophenolate mofetil; surgType, surgery type; priDose, prior TAC dose; priC_0_, prior trough concentration; posALB, first postoperative albumin; ALB_NN, nearest neighbor albumin; HB_NN, nearest neighbor hemoglobin; CR_NN, nearest neighbor creatinine; TB_NN, nearest neighbor total bilirubin; CYP3A5, genotype).

Based on minimal variables, the next step should be to identify and select final features that are suitable for clinical usage. Statistical histograms can visually analyze the cumulative frequency of candidate variables. Figure 5 shows a comprehensive frequency histogram for these combinations. It presents the frequency distribution of selected features across all variable combinations based on candidate variables by ten calculations. In M1, weight, MMF, and CYP3A5 were highly frequent (>35). In M2, five variables (priDose, priC_0_, weight, TB_NN, and CYP3A5) showed a significantly higher frequency than other factors. Additionally, posALB, posTB, and CR_NN were excluded due to low selection frequency (≤5).

**FIGURE 6:**
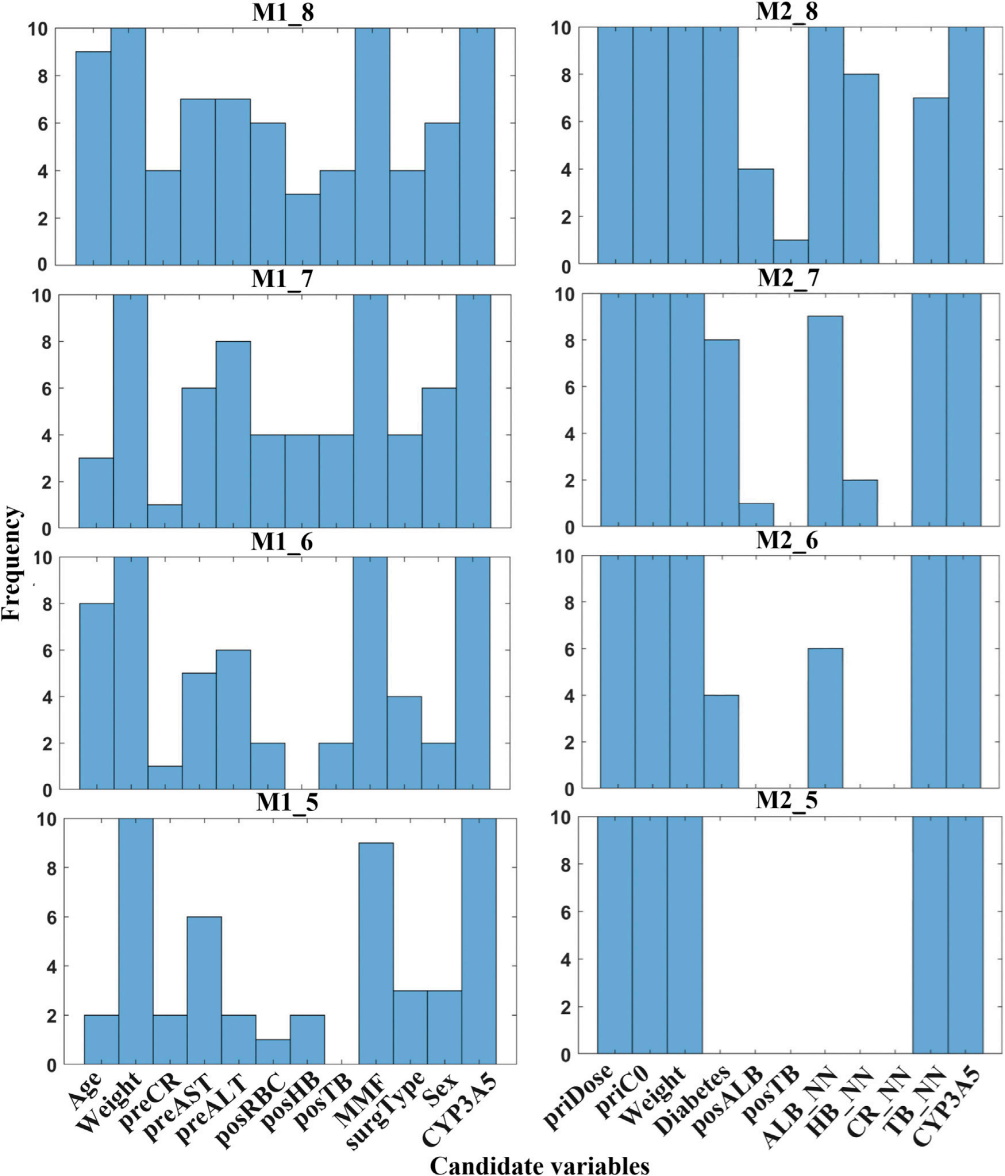
Frequency histogram of RBFSVR-GA-selected variables across individual combination in both models. (preCR, last preoperative creatinine; preALT, last preoperative alanine aminotransferase; preAST, last preoperative aspartate aminotransferase; posRBC, first postoperative red blood cells; posHB, first postoperative hemoglobin; posTB, first postoperative total bilirubin; MMF, mycophenolate mofetil; surgType, surgery type; priDose, prior TAC dose; priC_0_, prior trough concentration; posALB, first postoperative albumin; ALB_NN, nearest neighbor albumin; HB_NN, nearest neighbor hemoglobin; CR_NN, nearest neighbor creatinine; TB_NN, nearest neighbor total bilirubin; CYP3A5, genotype).

Figure 6 displays the subdivided frequency histogram of the remaining variables. In M1, potential variables included preCR, preALT, preAST, posRBC, posHB, posTB, surgType, and sex. In M2, potential variables were diabetes and ALB_NN. According to clinical knowledge, preCR, preALT, posHB, and posTB were added to M1 and ALB_NN was added to M2. Table 5 presents the reduced set of clinically applicable key variables for dose prediction in both models. Both models identified body weight and CYP3A5 genotype as critical factors of TAC dosing, aligning with established pharmacokinetic principles (Stratta et al., 2012). Further discussion will follow in the next section.

**TABLE 5:** EFS-DF-selected clinical applicable variables in both models.

Models	Clinical variables
Initial dose prediction	Weight, preCR, preALT, posHB, posTB, MMF, CYP3A5
Follow-up dose prediction	priDose, priC_0_, weight, ALB_NN, TB_NN, CYP3A5

preCR, last preoperative creatinine; preALT, last preoperative alanine aminotransferase; posHB, first postoperative hemoglobin; posTB, first postoperative total bilirubin; MMF, mycophenolate mofetil; priDose, prior TAC, dose; priC_0_, prior trough concentration; ALB_NN, nearest neighbor albumin; TB_NN, nearest neighbor total bilirubin; CYP3A5, genotype.

### 3.4 Comprehensive comparison of variables and algorithms

The original variables are all collected variables, candidate variables are pre-selected by GA, and clinical variables are the final key variables selected through EFS and statistical analysis from candidate variables. As shown in Table 6, both models maintained comparable performance despite a >70% reduction in feature count. Figure 4 further demonstrates the robustness of this feature selection. Specifically, candidate variables in M1 achieved significantly higher *R* ^2^ values than original variables, while other metrics (Acc, MSE, and MAE) showed only marginal decreases in both models. Notably, the refined clinical variables maintained good performance and even exhibited enhancement on some measures. M1 showed significant improvements in key metrics through clinical variable optimization, achieving Acc = 0.845, MSE = 0.554, MAE = 0.554, MEAE = 0.365, and *R* ^2^ = 0.603. In M2, only Acc and ME showed significant differences versus candidate variables. This feature reduction not only enhanced prediction accuracy but also improved model interpretability, both of which are critically important for clinically practical applications (Schagen et al., 2023).

**TABLE 6:** Performance using different variables in both models.

Models	Variables	Num	Acc	MSE	MAE	MEAE	ME	*R* ^2^
Initial dose prediction	Original	23	0.810	0.576	0.572	0.428	1.891	0.475
	Candidate	12	0.810	0.556	0.569	0.419	1.869	0.500
	Clinical	7	0.845	0.554	0.554	0.365	1.886	0.603
Follow-up dose prediction	Original	28	0.906	0.600	0.584	0.449	2.348	0.835
	Candidate	11	0.906	0.557	0.576	0.460	2.414	0.847
	Clinical	6	0.917	0.586	0.584	0.493	2.543	0.839

Furthermore, seven common machine learning methods were compared: LR, SVR, RF, ET, AdaBoost, GBDT, and LightGBM. As shown in Table 7, the average performance achieved in M1 was Acc = 0.795, MSE = 0.612, MAE = 0.598, MEAE = 0.421, ME = 1.970, and *R* ^2^ = 0.542, while M2 achieved Acc = 0.889, MSE = 0.671, MAE = 0.620, MEAE = 0.502, ME = 2.576, and *R* ^2^ = 0.814. These results confirm the effectiveness of our clinical variable selection method. Notably, RF, GBDT, and DF outperformed the average in both models. The DF algorithm demonstrated particularly strong performance, achieving Acc = 0.845/*R* ^2^ = 0.603 for initial dose prediction and Acc = 0.917/*R* ^2^ = 0.839 for follow-up prediction. Using fewer clinical factors, the cascaded ensemble learning approach delivered superior prediction performance.

**TABLE 7:** Performance using different algorithms based on the final factors in both models.

Models	Algorithms	Acc	MSE	MAE	MEAE	ME	*R* ^2^
Initial dose prediction	LR	0.759	0.633	0.589	0.439	2.160	0.504
	SVR	0.793	0.611	0.608	0.457	2.366	0.531
	RF	0.828	0.586	0.599	0.485	1.840	0.562
	ET	0.759	0.668	0.605	0.355	2.030	0.459
	AdaBoost	0.810	0.619	0.633	0.468	1.675	0.520
	GBDT	0.793	0.558	0.559	0.342	1.905	0.598
	LightGBM	0.776	0.665	0.639	0.460	1.898	0.562
	DF	0.845	0.554	0.554	0.365	1.886	0.603
	Mean	0.795	0.612	0.598	0.421	1.970	0.542
Follow-up dose prediction	LR	0.885	0.812	0.618	0.419	3.129	0.777
	SVR	0.896	0.604	0.605	0.431	2.402	0.834
	RF	0.890	0.640	0.623	0.515	2.501	0.824
	ET	0.867	0.685	0.649	0.618	2.338	0.812
	AdaBoost	0.875	0.763	0.684	0.575	2.590	0.791
	GBDT	0.906	0.545	0.544	0.441	2.433	0.831
	LightGBM	0.875	0.729	0.649	0.524	2.668	0.800
	DF	0.917	0.586	0.584	0.493	2.543	0.839
	Mean	0.889	0.671	0.620	0.502	2.576	0.814

## 4 Discussion

TAC is the most commonly used immunosuppressant for preventing acute rejection after kidney transplant (Kasiske et al., 2010). Clinically, deviations outside the narrow therapeutic window can cause significant adverse effects including diabetes, hypertension, nephrotoxicity, neurotoxicity, and opportunistic infections. Long-term under- or overexposure to TAC puts recipients at great risk of death, and it increasingly requires precise management (Brunet et al., 2019). However, an algorithm-based approach holds great potential to solve this issue of personalized drug administration (Schagen et al., 2023; Blasiak et al., 2020). The current study proposes an algorithmic pipeline to optimize variable selection for the clinically oriented dose prediction of TAC. It yielded an average accuracy of 84.5% for the starting dose and 91.7% for the follow-up dose. Likewise, the fitting-effect index *R* ^2^ reached 0.603 and 0.839, respectively. This result was superior to a recent study that used a deep learning algorithm to predict follow-up doses of TAC in kidney transplants (Zhang et al., 2022), which yielded *R* ^2^ of 82.4% with 25 clinical factors. For the initial dose, Table 8 presented a calculator for popPK analysis of the Chinese population, indicating that the proposed algorithm based on GA-DF performed better on the same testing set. The cascaded deep forest achieved optimal prediction using the selected feature factors. Its cascade architecture and ensemble learning enable powerful data representation with minimal hyperparameters and features. Its base evaluator was composed of trees in Figure 3. Tree-based approaches can offer enhanced interpretability, facilitating reliable clinical decision-making (Alzubaidi et al., 2021). Under the unique settings in the clinical application of kidney transplant, the GA-DF algorithm offers superior predictive performance.

**TABLE 8:** Comparison of initial dose prediction with previous study in the Chinese population.

Algorithm	Acc (%)	*R* ^2^
Dose (Ling et al., 2020) = 23.3×[HCT/0.309]^−0.445^ × [0.897, if POD>10] or [1, if POD≤10] × [1, if CYP3A5*1/*1 or CYP3A5*1/*3] or [0.657, if CYP3A5*3/*3] × 0.35	72.7	0.476
Our proposed method	84.5	0.603

HCT, hematocrit; POD, post-operative days; CYP3A5, genotype.

The clinical features selected by GA-EFS and input into the DF model accurately predicted actual dosing requirements. This predictive capability stems from the synergistic integration of global optimization and local fine-search strategies (Oh et al., 2004; Kato et al., 2018) which effectively identified clinically critical variables. Figure 4 shows that significant differences emerged during feature reduction: in initial dose prediction between seven and six variables (p < 0.05), and in follow-up dose prediction between six and five (p < 0.05). Therefore, for the prediction of initial doses, we ultimately selected seven variables, whereas for follow-up dose prediction, six proved sufficient. Under this setting, the EFS was further employed to evaluate the predictive performance of all fixed-number feature sets , and a histogram was used for statistical analysis (Figures 5, 6). Variables demonstrating both high frequency and clinical applicability are retained. Table 6 demonstrates robust model performance despite a 70% reduction in clinical features for both M1 and M2 compared to the original variables. Critically, the selected features align with TAC pharmacokinetic principles (Stratta et al., 2012; Iwasaki, 2007).

**FIGURE 7:**
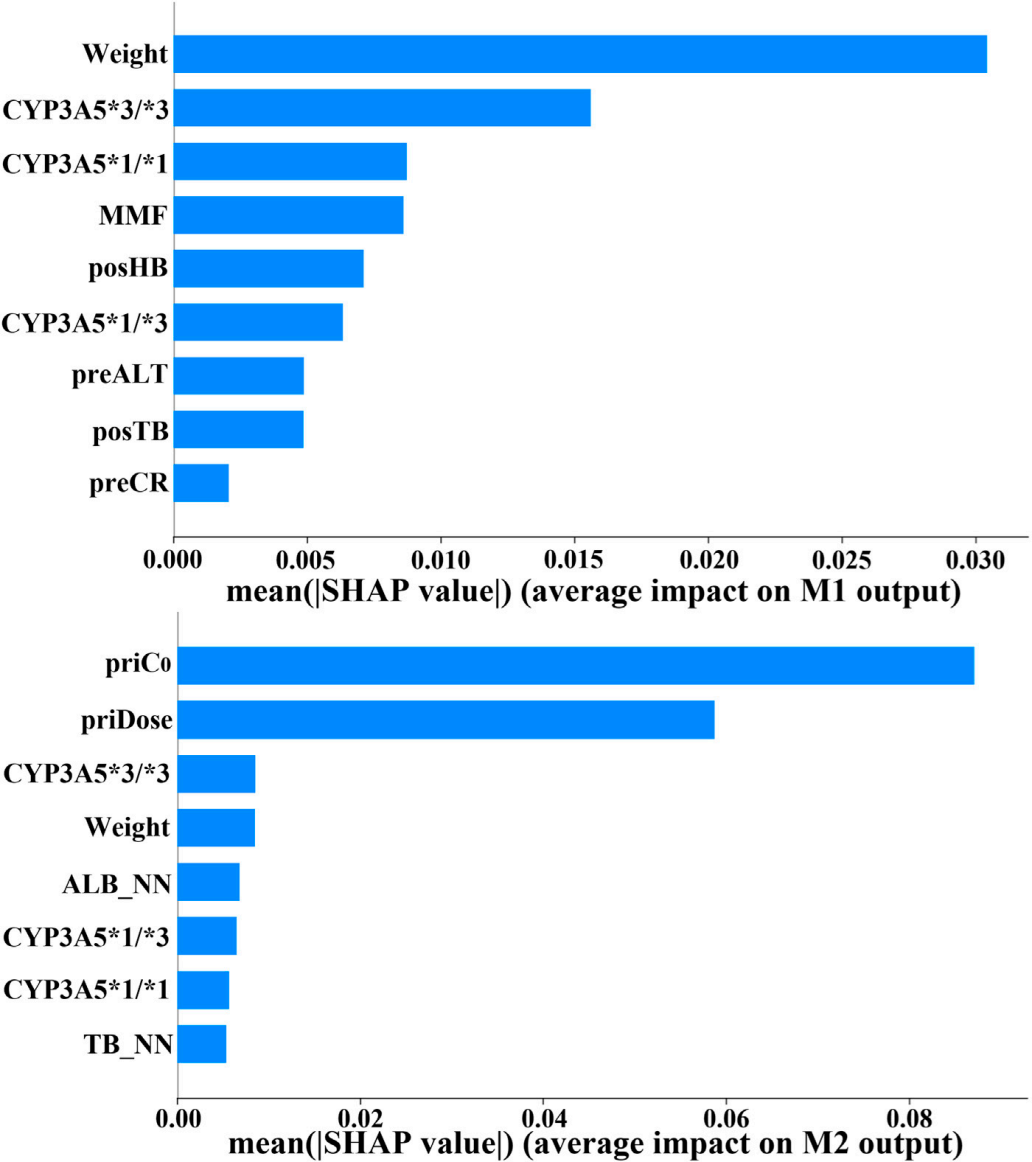
Feature importance ranking for clinical variables in initial/follow-up dose prediction models (CYP3A5, genotype; MMF, mycophenolate mofetil; posHB, first postoperative hemoglobin; preALT, last preoperative alanine aminotransferase; posTB, first postoperative total bilirubin; preCR, last preoperative creatinine; priC_0_, prior trough concentration; priDose, prior TAC dose; ALB_NN, nearest neighbor albumin; TB_NN, nearest neighbor total bilirubin).

**FIGURE 8:**
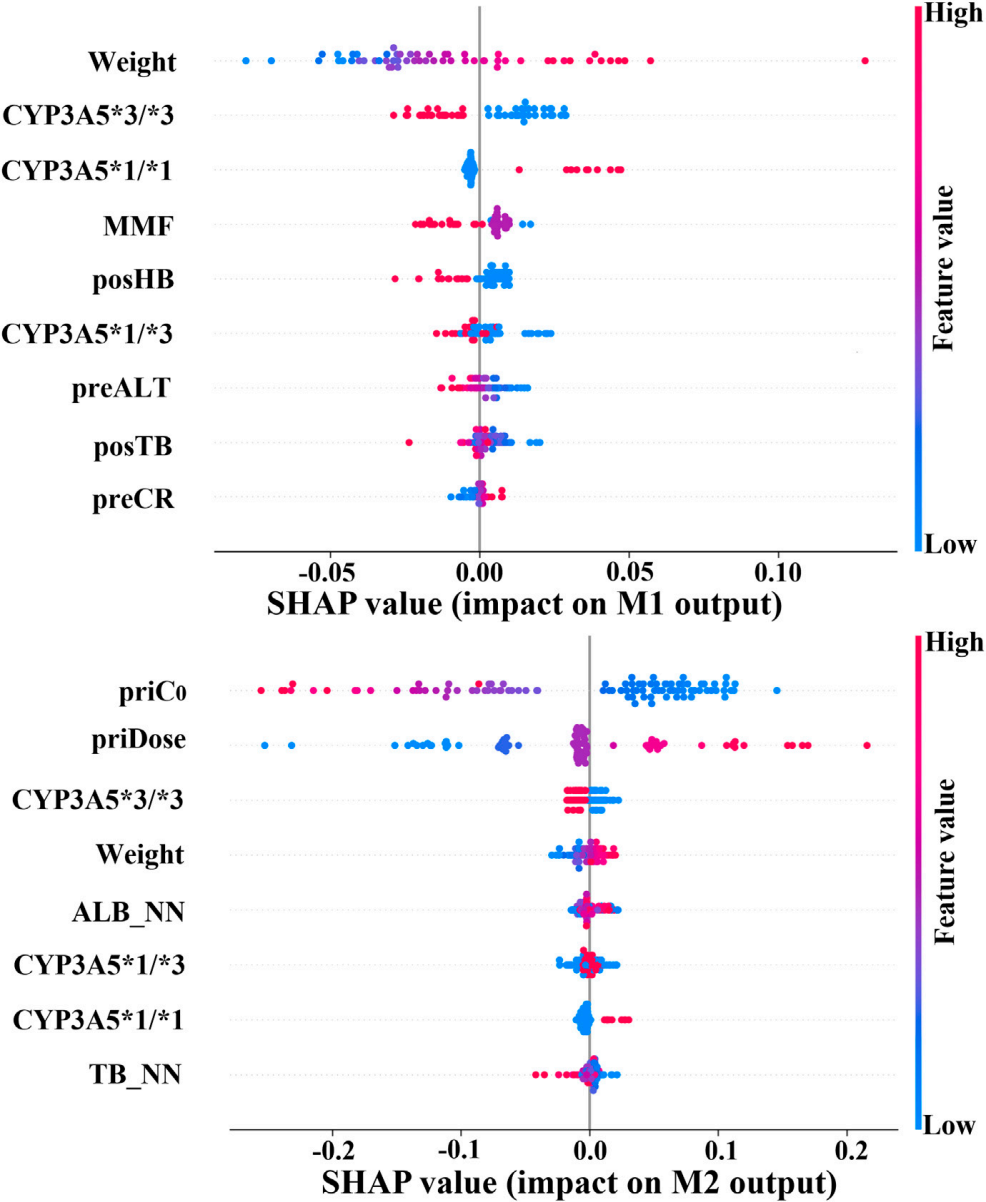
Output impact of clinical variables on initial/follow-up dose prediction models. Red dots on right: high feature values indicate positive contribution to model output. Blue dots on right: lower feature values correspond to positive contribution. (CYP3A5, genotype; MMF, mycophenolate mofetil; posHB, first postoperative hemoglobin; preALT, last preoperative alanine aminotransferase; posTB, first postoperative total bilirubin; preCR, last preoperative creatinine; priC_0_, prior trough concentration; priDose, prior TAC dose; ALB_NN, nearest neighbor albumin; TB_NN, nearest neighbor total bilirubin).

To validate this alignment, we utilized SHAP to quantify the contribution of clinical features within the prediction model (Winter 2002). Top variables that contributed the most to M1 and M2 outputs are displayed in Figure 7. Notably, CYP3A5 and body weight emerged as critical factors for TAC dosage prediction in both models. In fact, the CYP3A5 genotype has a significant effect on the metabolism of TAC reported in several studies (De Jonge et al., 2012; Renders et al., 2007; Barry and Levine, 2010; Yu et al., 2018). The *1/*1 is a fast metabolism that should increase the dose; the *3/*3 is a slow metabolism that should decrease the dose; the *1/*3 shows a neutral metabolism. Figure 8 shows full consistency in the performance of both M1 and M2 outputs, where right-positioned red dots indicate features with increased values that positively contribute to predicted dose while left-positioned blue dots correspond to features with negative contributions. Red points representing CYP3A5*1/*1 appear on the right (positive contribution), whereas blue points for CYP3A5*3/*3 cluster on the left (negative contribution). In addition, higher body weight is consistently correlated with an increased dosage requirement, establishing weight as a key reference for clinical dosing decisions (Mo et al., 2022). There is a positive correlation between body weight and required dosage in both models (right-positioned red dots). Our findings also verify that the TAC-MMF combination regimens enabled TAC dose reduction in the initial model (Eckhoff et al., 1998). Owing to TAC mainly being distributed in red blood cells, a higher HB can lead to a lower clearance rate, thereby allowing for a suitable TAC dosage reduction (Christians et al., 2002). Similarly, increased ALT indicates damaged or diseased liver, which can lead to a lower clearance of TAC. There is also alignment with clinical contexts. In particular, the prior dose and its corresponding C_0_ have a large influence on M2 output (Figures 7, 8), indicating the importance of prior TAC dosage.

Despite encouraging results from the proposed algorithm, particularly in follow-up dose prediction, several limitations warrant careful consideration in future research. The absence of external validation cohorts raises concern about model generalizability across diverse patients. Furthermore, the lack of prospective clinical assessment and formal evaluation of therapeutic outcomes such as rejection rates, toxicity reduction, or infection profiles prevents the use of the algorithm in real-world clinical practice. In order to achieve clinicians’ acceptance for predicting the initial/follow-up dose with a model, the developed models need to be externally validated and tested in a clinical trial. The utilization of algorithms requires the cooperation of professional clinicians. It can be integrated into a specialized database for kidney transplant systems or online prediction platforms. After recipients complete relevant clinical examinations, clinicians can directly retrieve the electronic records via patient ID and carry out initial or follow-up dose prediction. The generated dose recommendation should be combined with the clinical judgment of clinicians, who will make the final dosing decision. Both algorithm- and clinician-based dose will be logged simultaneously into a structured table for subsequent therapeutic efficacy assessment and model refinement/update. A good user interface is conducive to conducting prospective validation and can also collect useful outcomes for clinicians. In addition, this algorithm is specifically designed for the adult Chinese population, and the sample size is relatively small, especially for the initial dose data. The CYP3A5 genotype frequencies demonstrate a significant interethnic variation (Roy et al., 2005). Diverse geographic and ethnic populations, including pediatric recipients with a larger sample pool in multi-center or international cohorts, should be studied in the future. Despite the challenges, algorithm-based models for personalized TAC dosing can achieve precise dosing and reduce repetitive work and will thus receive increasing attention.

## 5 Conclusion

Kidney transplants are the most effective treatment for patients with end-stage renal disease. Long-term use of the immunosuppressive TAC is required after kidney transplant. However, there is high variability and a narrow therapeutic window. Traditional TDM is inadequate for personalized dosing. A novel clinical-oriented dosing algorithm in kidney transplants is proposed to explore important clinical variables and forecast the starting/follow-up doses. Utilizing a genetic algorithm (GA), we performed global optimization to identify candidate variables, followed by statistically rigorous exhaustive feature selection (EFS) to refine clinically significant factors. The analysis identified body weight, CYP3A5, CR, ALT, HB, TB, and MMF as significant predictors for initial dosing. Conversely, prior dose, prior C_0_, body weight, CYP3A5, ALB, and TB emerged as clinically critical factors for follow-up dosing. These findings were consistent with the pharmacokinetics of TAC reported in previous literature. The optimal prediction performance achieved by deep forest (DF) had an average Acc of 84.5% and *R* ^2^ of 0.603 for the former, with 91.7% and 0.839 the latter, respectively. The proposed methodology can be potentially extended to additional therapeutic areas requiring algorithm-based dose prediction.

## Data Availability

The raw data supporting the conclusions of this article will be made available by the authors, without undue reservation.

## References

[B1] Abdalla S. Abd-Allah F. Abdel Aziz M. (2015). Global, regional, and national age-sex specific all-cause and cause-specific mortality for 240 causes of death, 1990-2013: a systematic analysis for the global burden of disease study 2013. 10.1016/S0140-6736(14)61682-2 PMC4340604 25530442

[B2] Alzubaidi L. Zhang J. Humaidi A. J. Al-Dujaili A. Duan Y. Al-Shamma O. (2021). Review of deep learning: concepts, CNN architectures, challenges, applications, future directions. J. big Data 8, 53–74. 10.1186/s40537-021-00444-8 33816053 PMC8010506

[B3] Andrews L. M. Hesselink D. A. van Gelder T. Koch B. C. P. Cornelissen E. A. M. Brüggemann R. J. M. (2018). A population pharmacokinetic model to predict the individual starting dose of tacrolimus following pediatric renal transplantation. Clin. Pharmacokinet. 57, 475–489. 10.1007/s40262-017-0567-8 28681225 PMC5856873

[B4] Axelrod D. A. Schnitzler M. A. Xiao H. Irish W. Tuttle-Newhall E. Chang S. H. (2018). An economic assessment of contemporary kidney transplant practice. Am. J. Transplant. 18 (5), 1168–1176. 10.1111/ajt.14702 29451350

[B6] Barry A. Levine M. (2010). A systematic review of the effect of CYP3A5 genotype on the apparent oral clearance of tacrolimus in renal transplant recipients. Ther. drug Monit. 32 (6), 708–714. 10.1097/FTD.0b013e3181f3c063 20864901

[B7] Blasiak A. Khong J. Kee T. (2020). CURATE. AI: optimizing personalized medicine with artificial intelligence. SLAS Technol. Transl. Life Sci. Innov. 25 (2), 95–105. 10.1177/2472630319890316 31771394

[B8] Brunet M. van Gelder T. Åsberg A. Haufroid V. Hesselink D. A. Langman L. (2019). Therapeutic drug monitoring of tacrolimus-personalized therapy: second consensus report. Ther. drug Monit. 41 (3), 261–307. 10.1097/FTD.0000000000000640 31045868

[B9] Cai N. Zhang X. Zheng C. Zhu L. Zhu M. Cheng Z. (2020). A novel random forest integrative approach based on endogenous CYP3A4 phenotype for predicting tacrolimus concentrations and dosages in Chinese renal transplant patients. J. Clin. Pharm. Ther. 45 (2), 318–323. 10.1111/jcpt.13074 31721244

[B10] Choshi H. Miyoshi K. Tanioka M. Arai H. Tanaka S. Shien K. (2025). Long short-term memory algorithm for personalized tacrolimus dosing: a simple and effective time series forecasting approach post-lung transplantation. J. Heart Lung Transplant. 44 (3), 351–361. 10.1016/j.healun.2024.10.026 39510206

[B11] Christians U. Jacobsen W. Benet L. Z. Lampen A. (2002). Mechanisms of clinically relevant drug interactions associated with tacrolimus. Clin. Pharmacokinet. 41, 813–851. 10.2165/00003088-200241110-00003 12190331

[B12] De Jonge H. de Loor H. Verbeke K. Vanrenterghem Y. Kuypers D. R. (2012). *In* vivo CYP3A4 activity, CYP3A5 genotype, and hematocrit predict tacrolimus dose requirements and clearance in renal transplant patients. Clin. Pharmacol. and Ther. 92 (3), 366–375. 10.1038/clpt.2012.109 22871995

[B13] Diez-Sanmartin C. Sarasa-Cabezuelo A. Belmonte A. A. (2021). The impact of artificial intelligence and big data on end-stage kidney disease treatments. Expert Syst. Appl. 180, 115076. 10.1016/j.eswa.2021.115076

[B14] Eckhoff D. E. McGuire B. M. Frenette L. R. Contreras J. L. Hudson S. L. Bynon J. S. (1998). Tacrolimus (fk506) and mycophenolate mofetil combination therapy *versus* tacrolimus in adult liver transplantation. Transplantation 65 (2), 180–187. 10.1097/00007890-199801270-00006 9458011

[B15] Freund Y. Schapire R. E. (1996). “Experiments with a new boosting algorithm,” in Icml (Morgan Kaufmann, San Francisco: Citeseer).

[B16] Friedman J. H. (2001). Greedy function approximation: a gradient boosting machine. Ann. statistics 29, 1189–1232. 10.1214/aos/1013203451

[B17] Geurts P. Ernst D. Wehenkel L. (2006). Extremely randomized trees. Mach. Learn. 63, 3–42. 10.1007/s10994-006-6226-1

[B18] Hamet P. Tremblay J. (2017). Artificial intelligence in medicine. Metabolism 69, S36–S40. 10.1016/j.metabol.2017.01.011 28126242

[B19] Iwasaki K. (2007). Metabolism of tacrolimus (FK506) and recent topics in clinical pharmacokinetics. Drug metabolism Pharmacokinet. 22 (5), 328–335. 10.2133/dmpk.22.328 17965516

[B20] Kasiske B. L. Zeier M. G. Chapman J. R. Craig J. C. Ekberg H. Garvey C. A. (2010). KDIGO clinical practice guideline for the care of kidney transplant recipients: a summary. Kidney Int. 77 (4), 299–311. 10.1038/ki.2009.377 19847156

[B21] Kato E. R. R. de Aguiar Aranha G. D. Tsunaki R. H. (2018). A new approach to solve the flexible job shop problem based on a hybrid particle swarm optimization and random-restart hill climbing. Comput. and Industrial Eng. 125, 178–189. 10.1016/j.cie.2018.08.022

[B22] Ke G. Meng Q. Finley T. Wang T. Chen W. Ma W. (2017). Lightgbm: a highly efficient gradient boosting decision tree. Adv. neural Inf. Process. Syst. 30.

[B23] Kirubakaran R. Stocker S. L. Hennig S. Day R. O. Carland J. E. (2020). Population pharmacokinetic models of tacrolimus in adult transplant recipients: a systematic review. Clin. Pharmacokinet. 59, 1357–1392. 10.1007/s40262-020-00922-x 32783100

[B24] Kovesdy C. P. (2022). Epidemiology of chronic kidney disease: an update 2022. Kidney Int. Suppl. 12 (1), 7–11. 10.1016/j.kisu.2021.11.003 35529086 PMC9073222

[B25] Leardi R. Boggia R. Terrile M. (1992). Genetic algorithms as a strategy for feature selection. J Chemometr. 6 (5), 267–281. 10.1002/cem.1180060506

[B26] Liashchynskyi P. Liashchynskyi P. (2019). Grid search, random search, genetic algorithm: a big comparison for NAS. arXiv. 10.48550/arXiv.1912.06059

[B27] Ling J. Dong L. L. Yang X. P. Qian Q. Jiang Y. Zou S. L. (2020). Effects of CYP3A5, ABCB1 and POR* 28 polymorphisms on pharmacokinetics of tacrolimus in the early period after renal transplantation. Xenobiotica 50 (12), 1501–1509. 10.1080/00498254.2020.1774682 32453653

[B28] Lundberg S. M. Lee S.-I. (2017). A unified approach to interpreting model predictions. Adv. neural Inf. Process. Syst. 30.

[B29] Mo X. Chen X. Wang X. Zhong X. Liang H. Wei Y. (2022). Prediction of tacrolimus dose/weight-adjusted trough concentration in pediatric refractory nephrotic syndrome: a machine learning approach. Pharmacogenomics Personalized Med. 15, 143–155. 10.2147/PGPM.S339318 35228813 PMC8881964

[B30] Oh I.-S. Lee J.-S. Moon B.-R. (2004). Hybrid genetic algorithms for feature selection. IEEE Trans. pattern analysis Mach. Intell. 26 (11), 1424–1437. 10.1109/TPAMI.2004.105 15521491

[B31] Otabil M. K. (2019). Genetic polymorphisms affecting tacrolimus dose requirements in Ghanaian patients with end-stage renal disease. Accra: University of Ghana.

[B32] Patro S. Sahu K. K. (2015). Normalization: a preprocessing stage. arXiv, 20–22. 10.17148/iarjset.2015.2305

[B33] Pedregosa F. Varoquaux G. Gramfort A. Michel V. Thirion B. Grisel O. (2011). Scikit-learn: machine learning in python. J. Mach. Learn. Res. 12, 2825–2830. 10.5555/1953048.2078195

[B34] Radhakrishnan A. Kuppusamy G. Ponnusankar S. Mutalik S. (2021). Towards next-generation personalization of tacrolimus treatment: a review on advanced diagnostic and therapeutic approaches. Pharmacogenomics 22 (17), 1151–1175. 10.2217/pgs-2021-0008 34719935

[B35] Renders L. Frisman M. Ufer M. Mosyagin I. Haenisch S. Ott U. (2007). CYP3A5 genotype markedly influences the pharmacokinetics of tacrolimus and sirolimus in kidney transplant recipients. Clin. Pharmacol. and Ther. 81 (2), 228–234. 10.1038/sj.clpt.6100039 17192769

[B36] Roy J.-N. Lajoie J. Zijenah L. S. Barama A. Poirier C. Ward B. J. (2005). CYP3A5 genetic polymorphisms in different ethnic populations. Drug metabolism Dispos. 33 (7), 884–887. 10.1124/dmd.105.003822 15833928

[B37] Schagen M. R. Volarevic H. Francke M. I. Sassen S. D. T. Reinders M. E. J. Hesselink D. A. (2023). Individualized dosing algorithms for tacrolimus in kidney transplant recipients: current status and unmet needs. Expert Opin. Drug Metabolism and Toxicol. 19 (7), 429–445. 10.1080/17425255.2023.2250251 37642358

[B38] Staatz C. E. Tett S. E. (2004). Clinical pharmacokinetics and pharmacodynamics of tacrolimus in solid organ transplantation. Clin. Pharmacokinet. 43 (10), 623–653. 10.2165/00003088-200443100-00001 15244495

[B39] Staatz C. E. Willis C. Taylor P. J. Tett S. E. (2002). Population pharmacokinetics of tacrolimus in adult kidney transplant recipients. Clin. Pharmacol. and Ther. 72 (6), 660–669. 10.1067/mcp.2002.129304 12496747

[B40] Stifft F. Vandermeer F. Neef C. van Kuijk S. Christiaans M. H. L. (2020). A limited sampling strategy to estimate exposure of once-daily modified release tacrolimus in renal transplant recipients using linear regression analysis and comparison with Bayesian population pharmacokinetics in different cohorts. Eur. J. Clin. Pharmacol. 76, 685–693. 10.1007/s00228-019-02814-x 32020321

[B41] Stratta P. Quaglia M. Cena T. Antoniotti R. Fenoglio R. Menegotto A. (2012). The interactions of age, sex, body mass index, genetics, and steroid weight-based doses on tacrolimus dosing requirement after adult kidney transplantation. Eur. J. Clin. Pharmacol. 68, 671–680. 10.1007/s00228-011-1150-0 22101623

[B42] Tang J. Liu R. Zhang Y. L. Liu M. Z. Hu Y. F. Shao M. J. (2017). Application of machine-learning models to predict tacrolimus stable dose in renal transplant recipients. Sci. Rep. 7 (1), 42192. 10.1038/srep42192 28176850 PMC5296901

[B43] Van Looy S. Verplancke T. Benoit D. Hoste E. Van Maele G. De Turck F. (2007). A novel approach for prediction of tacrolimus blood concentration in liver transplantation patients in the intensive care unit through support vector regression. Crit. Care 11, R83–R87. 10.1186/cc6081 17655766 PMC2206504

[B44] Vincenti F. Jensik S. C. Filo R. S. Miller J. Pirsch J. (2002). A long-term comparison of tacrolimus (FK506) and cyclosporine in kidney transplantation: evidence for improved allograft survival at five years. Transplantation 73 (5), 775–782. 10.1097/00007890-200203150-00021 11907427

[B45] Winter E. (2002). The shapley value. Handb. game theory Econ. Appl. 3, 2025–2054. 10.1016/S1574-0005(02)03016-3

[B46] Yu M. Liu M. Zhang W. Ming Y. (2018). Pharmacokinetics, pharmacodynamics and pharmacogenetics of tacrolimus in kidney transplantation. Curr. drug Metab. 19 (6), 513–522. 10.2174/1389200219666180129151948 29380698 PMC6182932

[B47] Zhang Q. Tian X. Chen G. Yu Z. Zhang X. Lu J. (2022). A prediction model for tacrolimus daily dose in kidney transplant recipients with machine learning and deep learning techniques. Front. Med. 9, 813117. 10.3389/fmed.2022.813117 35712101 PMC9197124

[B48] Zhou Z.-H. Feng J. (2019). Deep forest. Natl. Sci. Rev. 6 (1), 74–86. 10.1093/nsr/nwy108 34691833 PMC8291612

[B49] Zhu W. Xue L. Peng H. Duan Z. Zheng X. Cao D. (2018). Tacrolimus population pharmacokinetic models according to CYP3A5/CYP3A4/POR genotypes in Chinese Han renal transplant patients. Pharmacogenomics 19 (13), 1013–1025. 10.2217/pgs-2017-0139 30040022

